# A percutaneous treatment strategy of an adult patient with a bicuspid aortic valve, coarctation of the aorta, and an exceptionally large aneurysm of a collateral artery: Case report and literature overview

**DOI:** 10.3389/fcvm.2022.1012147

**Published:** 2022-12-22

**Authors:** D. B. H. Verheijen, J. Lauran Stöger, F. van der Kley, M. J. Schalij, M. R. M. Jongbloed, H. W. Vliegen, P. Kiès, A. D. Egorova

**Affiliations:** ^1^CAHAL, Center for Congenital Heart Disease Amsterdam Leiden, Leiden University Medical Center, Leiden, Netherlands; ^2^Department of Cardiology, Leiden University Medical Center, Leiden, Netherlands; ^3^Department of Radiology, Leiden University Medical Center, Leiden, Netherlands; ^4^Department of Anatomy and Embryology, Leiden University Medical Center, Leiden, Netherlands

**Keywords:** coarctation of aorta, bicuspid aortic valve, collateral arteries, aneurysm, endocarditis, percutaneous intervention, percutaneous catheter technique, adult congenital heart disease

## Abstract

Coarctation of the aorta (CoA) is a congenital heart defect that is associated with a bicuspid aortic valve (BAV), ascending aorta dilatation, intracerebral aneurysms, and premature atherosclerotic disease. The first presentation during late adulthood is rare and is frequently driven by late sequelae. Hypertrophic collateral arteries can develop aneurysms which are at risk for spontaneous rupture, however, treatment recommendations for these aneurysms are scarce. Here, we describe the clinical course and percutaneous treatment strategy of a patient with a late diagnosis of a pin-point CoA, a BAV with moderate regurgitation, and an exceptionally large aneurysm of a collateral artery. A 59-year-old woman was diagnosed with *Streptococcus bovis* endocarditis of a BAV with moderate aortic valve regurgitation and small vegetation (<5 mm) on the non-coronary cusp. Work-up revealed hypertension and adenocarcinoma *in situ* of the ascending colon, considered the bacteremia porte d’entrée, for which a curative hemicolectomy was performed. Echocardiography showed a narrowing of the aorta distal from the origin of the left subclavian artery with the antegrade diastolic flow with a pathognomonic “sawtooth” pattern and an estimated pressure gradient of >70 mmHg. Computed tomography angiography (CTA) showed a network of well-developed collateral arteries and a levoatriocardinal vein. One of the collateral arteries arising from the left subclavian artery revealed an exceptionally large aneurysmatic dilation (29 × 24 × 24 mm). The invasive assessment confirmed a hemodynamically significant CoA. Treatment involved balloon dilatation and placement of a covered stent at the site of the pin-point CoA and a percutaneous coronary intervention (PCI) of the stenosis in the left anterior descending artery. No residual gradient over the CoA was observed. Antihypertensive drugs could be discontinued, and CTA performed 4 months later showed regression and thrombosis of the numerous collaterals and, importantly, thrombosis of the large aneurysm. This case illustrates the late diagnosis of CoA with associated congenital heart defects and late sequelae including hypertension, BAV endocarditis, coronary artery disease, and aneurysm formation of the extensive collateral network. The patient underwent pharmacological and percutaneous treatment, ultimately resulting in the alleviation of the CoA, normalization of the blood pressure, reduction of collateral flow, and thrombosis of the large aneurysm of the collateral artery.

## Introduction

Coarctation of the aorta (CoA) is a relatively common congenital heart defect, accounting for 5–8% of all congenital heart and great vessel anomalies and is reported in about 1–2,500 of live births with a male preponderance (1–4). Narrowing of the aorta in the region of the ductus/ligamentum arteriosus (often referred to as juxtaductal or paraductal) is the most frequently diagnosed CoA in adults (5, 6). CoA can present as an isolated defect, however, it is often associated with other cardiac defects, including a bicuspid aortic valve in up to 85% of patients and intracerebral aneurysms of the circle of Willis in up to 10% of cases (2, 3, 7–10). Arterial hypertension is usually present, and CoA is associated with an increased risk of aortic dilatation and aneurysm formation (particularly ascending aorta or isthmus), premature coronary artery disease (CAD), and infective endocarditis or endarteritis (10–13).

Clinical presentation of CoA largely depends on the hemodynamic severity of the flow limitation in the thoracic aorta. During the neonatal period, presentation is usually related to left ventricular dysfunction—due to increased afterload after the closure of the ductus arteriosus—and decreased oxygen saturation due to a limitation of blood supply to the lower extremities (3). In adulthood, patients more often present with morbidity caused by prolonged upper extremity hypertension or late vascular complications (3). Untreated or undiagnosed adults with sub-clinical CoA typically have either a relatively mild stenosis or sufficiently developed collateral arteries. Treatment-resistant hypertension or a murmur may have been documented, but are often not recognized as the sequelae of a CoA, contributing to delayed diagnosis.

A blood pressure difference between the upper and lower extremities of ≥20 mmHg, a radio-radial or radio-femoral delay, and weak or absent arterial pulses in the lower extremities suggest a significant hemodynamic CoA. In the presence of a sufficiently developed collateral network bypassing the coarctation, the *trans*-coarctation pressure gradient is often less severe (2, 14). Transthoracic echocardiography is usually the first technique to diagnose CoA or to at least raise the suspicion thereof. Cardiovascular magnetic resonance imaging (MRI) and computed tomography angiography (CTA) are better suited to assess the anatomy of the aorta and the isthmus in detail, in particular, the spatial relation between the CoA and the surrounding (collateral) arteries. For the evaluation of the hemodynamic significance of the CoA, an invasive hemodynamic assessment is imperative, with a peak-to-peak gradient ≥20 mmHg being considered significant (15, 16).

In adults, the preferred management strategy of CoA is the implantation of a covered stent by a percutaneous endovascular intervention (17–19). However, treatment recommendations for associated defects such as collateral arterial aneurysms are more scarce. This case report describes the clinical course and treatment strategy of a patient with a moderate regurgitation of a bicuspid aortic valve, a mildly dilated ascending aorta, and a hemodynamically significant focal CoA with an exceptionally large aneurysm of a collateral artery arising from the left subclavian artery.

## Case presentation

A 59-year-old woman with no medical history apart from hypercholesterolemia at the time of presentation presented to the emergency department with complaints of malaise and a persistent fever of >39°C. She reported fatigue, unintentional weight loss, and night sweats in the months prior to the presentation. She was hypertensive with an upper extremity blood pressure of 160/65 mmHg, heart rate of 116 bpm, and temperature of 39.7°C. Auscultation revealed a grade II/VI diastolic and grade III/VI systolic murmur with punctum maximum over the aortic valve and a loud continuous murmur over the left scapula. No signs of congestion or endocarditis stigmata were documented. She was admitted and empirically treated with flucloxacillin, which was switched to benzylpenicillin when multiple blood cultures showed the growth of *Streptococcus bovis* within the first 24 h. Further abdominal imaging (performed due to the known association with colon tumors of a *Streptococcus bovis bacteremia*) revealed an adenocarcinoma *in situ* of the ascending colon, which was considered the porte d’entrée. The patient was deemed eligible for curative hemicolectomy with stage T1N0M0 malignancy.

**FIGURE 1:**
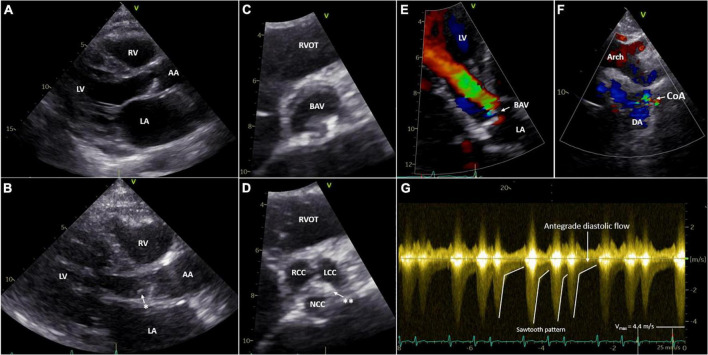
Transthoracic echocardiography of the aortic valve **(A–E)** and CoA **(F,G)**. Parasternal long-axis view of the aortic valve open **(A)** and closed with a mobile mass on the non-coronary cusp **(B)**. Parasternal short-axis view of BAV open **(C)** and closed **(D)**. Apical five-chamber view of the BAV with moderate aortic regurgitation with color doppler **(E)**. Suprasternal view of the aortic arch and CoA with color doppler **(F)**. Sawtooth pattern on echo doppler of the CoA **(G)**. LV, left ventricle; RV, right ventricle; LA, left atrium; AA, ascending aorta; DA, descending aorta; RVOT, right ventricular outflow tract; BAV, bicuspid aortic valve; RCC, right coronary cusp; LCC, left coronary cusp; NCC, non-coronary cusp; Arch, aortic arch; CoA, coarctation of the aorta, *Mobile mass; ^**^raphe between the NCC and LCC.

**FIGURE 2:**
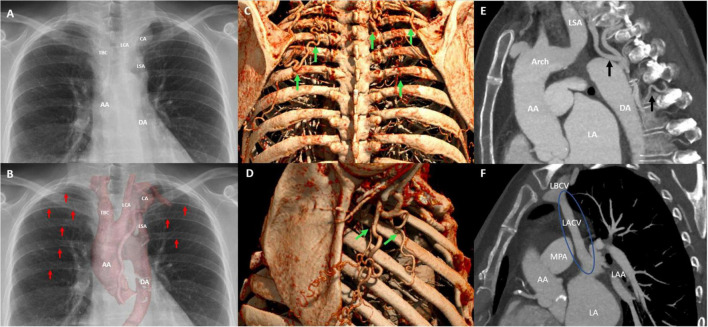
Postero-anterior chest radiograph illustrates the aneurysmatic collateral artery projecting over the apical–medial aspect left upper lobe and mediastinal silhouette **(A)**. In addition, the postero-anterior chest radiograph with multifocal rib notching (red arrows) and projection of the cinematic 3D-reconstruction of the thoracic aorta illustrating the CoA and associated aneurysmatic collateral artery **(B)**. Oblique sagittally reformatted maximum intensity projection (MIP) cinematic 3D-rendering of thoracic CTA shows hypertrophic collateral arteries posterior **(C)** and lateral **(D)** alongside the chest wall, indicated by green arrows. The sagittal CTA image illustrating collateral arteries and hypertrophic intercostal arteries inserting into the descending aorta **(E)**, and the levoartriocardinal vein **(F)**. AA, ascending aorta; DA, descending aorta; CA, aneurysmatic collateral artery; LSA, left subclavian artery; LCA, left carotid artery; TBC, truncus brachiocephalicus; Arch, aortic arch; LSA, left subclavian artery; LA, left atrium; LACV, levoatriocardinal vein; LBCV, left brachiocephalic vein.

Clinical work-up for a complicated bacteriemia, as well as the abnormalities on physical examination, prompted further evaluation. Transthoracic echocardiography showed a concentric hypertrophic left ventricle (LV) with an increased LV mass index (143 g/m^2^) with a good systolic function (LVEF 59%) and a functionally bicuspid aortic valve with moderate regurgitation and a mobile mass of 4 mm in length on the non-coronary cusp. Moreover, mild dilation of the ascending aorta (38 mm) and narrowing of the aorta distal from the origin of the left subclavian artery with the antegrade diastolic flow in the thoracic aorta with a typical “saw tooth” pattern and an estimated pressure gradient of 78 mmHg (Vmax 4.4 m/s) was observed, indicating significant aortic coarctation (Figures 1A–G). The ECG showed sinus rhythm with high voltages of the QRS complex in left precordial leads, meeting the Sokolow–Lyon criteria for left-ventricular hypertrophy. A chest X-ray showed subtle bilateral notching on the inferior borders of several posterior ribs and a rounded opacity projecting over the paramediastinal aspect of the left upper lobe (Figures 2A, B). Given the good response to antibiotic treatment, the small size of the vegetation (<10 mm), no signs of distal embolization, and non-severe aortic valve regurgitation which was hemodynamically well tolerated, conservative antibiotic treatment with intravenous benzylpenicillin for a total of 6 weeks was pursued after multidisciplinary evaluation by the endocarditis team (20–22). Blood cultures taken a day after antibiotic treatment initiation showed no growth, and the patient remained afebrile. After antibiotic treatment, the patient showed no signs of persistent or recurrent infection, and blood cultures 4 months following the last dose of benzylpenicillin remained negative.

**FIGURE 3:**
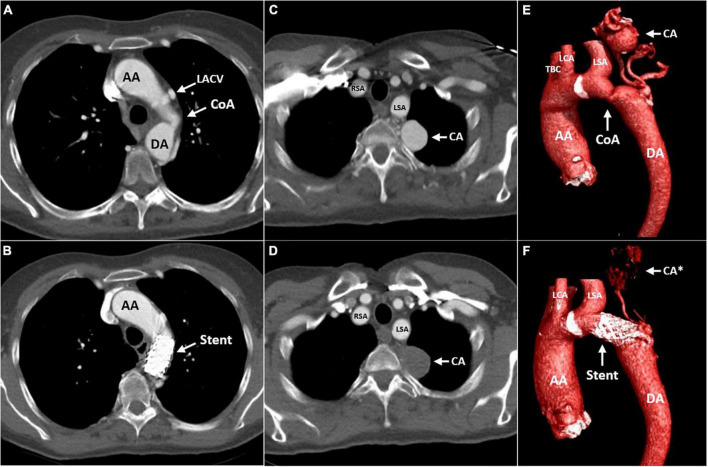
Computed tomography (CT) angiography of the aorta before **(A,C,E)** and 4 months after **(B,D,F)** implantation of a covered stent across the CoA. The transverse plane is at the level of the CoA **(A,B)** and the level of the left paravertebral aneurysmatic collateral artery **(C,D)**. Cinematic 3D reconstruction of the thoracic aorta, illustrating the CoA and associated aneurysmatic collateral artery **(E,F)**. AA, ascending aorta; DA, descending aorta; LACV, levoatriocardinal vein; CoA, coarctation of the aorta; Stent, covered stent in the coarctation zone; CA, aneurysmatic collateral artery; CA*, location of the aneurysmatic collateral artery; RSA, right subclavian artery; LSA, left subclavian artery; LCA, left carotid artery; TBC, truncus brachiocephalicus.

A CTA of the chest was performed to accurately visualize the anatomy of the thoracic aorta. This confirmed a severe, pin-point coarctation with a minimal double-oblique diameter of 4 × 4 mm just distal to the origin of the left subclavian artery. Furthermore, CTA revealed hypertrophic intercostal, internal mammary, and scapular arteries (Figures 2C–E), a levoatriocardinal vein (LACV) connecting the truncus brachiocephalicus with the left atrium (Figure 2F), and an extensive intrathoracic collateral network with a large paravertebral aneurysm (29 × 24 × 24 mm) of one of the collateral arteries arising from the left subclavian artery (Figures 3A, C, E). In addition, a high multi-vessel coronary plaque burden, including a significant proximal left anterior descending lesion, was documented. Given persistent hypertension, treatment with a dihydropyridine calcium channel blocker (amlodipine) and an angiotensin-converting enzyme inhibitor (lisinopril) was initiated.

**FIGURE 4:**
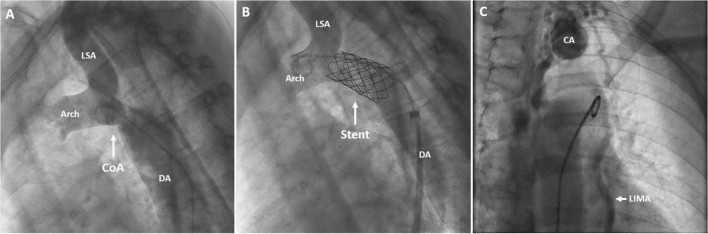
Angiography of the aortic arch and descending aorta before **(A)** and after stent implantation **(B)**. Angiography of the collateral artery aneurysm **(C)**. DA, descending aorta; CoA, coarctation of the aorta; Stent, covered stent in the coarctation zone; CA, collateral aneurysm; LSA, left subclavian artery; LIMA, left internal mammary artery; Arch, aortic arch.

A three-dimensional (3D) print of the heart, aorta, and collateral vessels, including the aneurysm, was constructed to facilitate a better understanding of the complex anatomy. The patient was discussed in the adult congenital heart team; considering the patient’s age and the perioperative risk of aortic surgery in the presence of extensive and aneurysmatic collaterals, we pursued a primarily percutaneous strategy. Invasive pressure measurements revealed systolic blood pressures proximal and distal to the CoA of 178 and 95 mmHg, respectively. This resulted in a gradient of 83 mmHg, warranting the indication for intervention. To treat the coarctation, a covered stent was implanted (Figures 4A, B) with good angiographic results and alleviation of the pressure gradient. Figure 4C shows the angiography of the collateral artery aneurysm that was subjected to conservative treatment. Coronary angiography confirmed a significant stenotic lesion in the proximal left anterior descending artery (80–90% stenosis), for which a percutaneous coronary intervention (PCI) with stent implantation was performed. The other coronary arteries showed non-significant atherosclerotic lesions. The post-operative period was uneventful, dual antiplatelet therapy with acetylsalicylic acid and clopidogrel was started, and amlodipine was discontinued. Two months later, losartan was discontinued due to the normalization of blood pressures to 100–125/70 mmHg.

Due to a reduced kidney function (creatinine 137 μmol/L, estimated glomerular filtration rate of 36 ml/min/1.73 m^2^) secondary to endocarditis-associated glomerulonephritis, statin therapy was not started immediately after PCI. Short-term treatment with oral prednisone was pursued by the IEAGN, and a statin could be initiated after the recovery of the renal function.

The patient was seen for a follow-up at the outpatient clinic 4 months after the procedure. She was normotensive and reported to be doing well, experiencing no functional limitations during daily activities. Transthoracic echocardiography showed stable dimensions of the ascending aorta, moderate insufficiency of the aortic valve, and preserved left ventricular function. The peak flow velocity over the coarctation was measured as 1.7 m/s, corresponding to a derived pressure gradient of 12 mmHg. CTA confirmed stable aortic dimensions and good position and deployment of the stent at the CoA site. The large collateral artery aneurysm no longer showed any contrast opacification, likely reflecting spontaneous thrombosis secondary to the diminished collateral flow after the alleviation of the coarctation (Figures 3B, D, F).

Furthermore, magnetic resonance angiography (MRA) was performed to screen for cerebral aneurysms that are often associated with CoA in adults. A small aneurysm in the proximal right anterior cerebral artery (A1 segment) with a maximum diameter of 2.5 mm was revealed. After 6 months, the MRA was repeated, showing stable dimensions and ensuing a conservative management strategy including periodic follow-up.

Currently, 20 months after the initial presentation, the patient is doing well with no signs of adenocarcinoma recurrence or anginal symptoms. Her blood pressure is 125/75 mmHg without antihypertensive medication. The patient remains under annual follow-up at our outpatient clinic.

## Discussion

Coarctation of the aorta is a congenital defect that may be caused by the constriction of the ductal tissue in the area of the ductus arteriosus, often associated with multiple other congenital heart anomalies and vascular comorbidities. Recognition of a CoA early in life is important to prevent potential complications since the natural course of an untreated CoA can lead to a plethora of long-term sequelae (23). Fortunately, hemodynamically important CoA is usually diagnosed prenatally or early after birth (23). However, unrecognized CoA can initially remain undiagnosed for many years, particularly when a sufficient collateral artery network is present (14, 24, 25). During adulthood, diagnosis is often made in the work-up of systemic hypertension or a heart murmur (2).

Here, we describe the case of a patient with a late diagnosis of CoA at the age of 59 in the setting of a late complication of an associated bicuspid aortic valve, namely *Streptococcus bovis* endocarditis.

The case is illustrative of several typical features of CoA. The patient’s ECG was suggestive of left ventricular hypertrophy, a consequence of the chronically increased afterload and systemic arterial hypertension. Transthoracic echocardiography revealed typical antegrade diastolic flow in the descending thoracic aorta with a “saw tooth” pattern (2, 26). In adults, the location opposite the ductus/ligamentum arteriosus is the most commonly encountered location of CoA (5, 6, 27). Hypertrophic intercostal, internal, mammary, and scapular arteries are illustrative of the typical compensatory collateral flow in adults with untreated CoA (7). The LACV is a rare pulmonary-systemic connection that most frequently connects the left atrium and the brachiocephalic veins (28). LACV predominantly presents in left-heart obstructive lesions (28–30). Several cases of LACV in the presence of CoA are described (28, 30–35). Bilateral posterior rib notching is a pathognomonic radiological phenomenon caused by tortuous dilation of the third to eighth intercostal arteries (36). As illustrated by this case, a bicuspid aortic valve can be diagnosed in up to 85% of patients with CoA (2, 3, 7–9). This patient presented with bacterial endocarditis as a late complication of a bicuspid valve (7). Long-standing and treatment-resistant upper extremity hypertension is an important yet often unrecognized physical finding in patients with CoA (2, 3).

Furthermore, CTA showed extensive coronary plaque burden. Although the patient’s age, pre-existent hypercholesterolemia, and former cigarette smoking contributed to the natural risk of atherosclerosis, the prevalence of premature CAD is increased in patients with CoA (37, 38). However, it is not clear whether CoA itself is an independent risk factor for CAD (37). In addition, studies show that increased cardiovascular risk does not directly decline to the level of the general population following interventional treatment of CoA (39). Therefore, secondary prevention is essential in preventing and halting the progression of CAD (40, 41). Although statin treatment in patients with CoA affects serum total cholesterol and LDL-cholesterol levels, it does not result in a lower risk of cardiovascular mortality or morbidity (42). Hence, targeting all the modifiable cardiovascular risk factors, including effective blood pressure regulation, remains highly important.

Although arterial aneurysms are frequently associated with CoA, they are seldom large. However, large aneurysms of collateral arteries are more prevalent in elderly patients with CoA (43). Several case reports demonstrate that, for instance, spinal and intercostal artery aneurysms are at risk for rupture, potentially with fatal outcomes (44). However, current guidelines fail to address the management of these aneurysms (45–47). Therefore, the treatment strategy was determined by the expert opinion of the multidisciplinary adult congenital heart disease team. Treatment options included surgical correction, percutaneous treatment (i.e., coiling), or re-evaluation after initial percutaneous treatment of the CoA. The rationale behind the latter was that alleviating the obstruction caused by the CoA would improve aortic hemodynamics and, in turn, diminish collateral blood flow. Subsequently, this could result in blood stasis in the aneurysmatic sac with spontaneous thrombosis. A similar treatment approach was previously described by Judicael and colleagues (48). However, in that case, blood flow in the aneurysm persisted (which was 33 mm in diameter), preventing spontaneous thrombosis despite successful treatment of the CoA. Complete exclusion of the aneurysm from the circulation was subsequently achieved by percutaneously delivered local injection of thrombin and glue. In our case, however, successful endovascular treatment of the CoA was sufficient to result in a reduction of collateral flow and thrombosis of the aneurysm, as illustrated by CTA imaging 4 months after the procedure.

In the described case, a CTA-based 3D print was constructed to improve the understanding of complex anatomy and spatial relations. Ultimately, this was helpful in supporting the treatment decision process and peri-procedural planning. In accordance with our experience, a systematic review by Lau et al. concluded that a 3D model improves the interpretation of the anatomy in patients with congenital heart disease (49). Furthermore, 3D-printing can be useful for preoperative planning and medical and patient education (49).

In conclusion, this case illustrates that CoA with multiple associated defects can remain undiagnosed until late adulthood in the presence of well-developed collateral arteries. As previously reported, one should be aware of CoA in adults with long-standing hypertension. Furthermore, this case demonstrates that a successful percutaneous management strategy of CoA can reduce collateral arterial flow, thus providing a valid treatment option for even exceptionally large collateral artery aneurysms.

## Data availability statement

The raw data supporting the conclusions of this article will be made available by the authors, without undue reservation.

## Ethics statement

All procedures performed involving the human participant were in accordance with the ethical standards of the institutional and/or national research committee and with the 1964 Helsinki Declaration and its later amendments or comparable ethical standards. The patient provided consent for the publication of this case report. Written informed consent was obtained from the individual(s) for the publication of any potentially identifiable images or data included in this article.

## Author contributions

DV drafted the manuscript. DV and JS created the figures. AE edited the manuscript. All authors were involved in the conceptualization, analysis, and interpretation of the diagnostic data and the management strategy described in the manuscript, and approved, and co-edited the manuscript for submission in its current form.
